# Diacylglycerol-dependent hexamers of the SNARE-assembling chaperone Munc13-1 cooperatively bind vesicles

**DOI:** 10.1073/pnas.2306086120

**Published:** 2023-10-26

**Authors:** Feng Li, Kirill Grushin, Jeff Coleman, Frederic Pincet, James E. Rothman

**Affiliations:** ^a^Department of Cell Biology, School of Medicine, Yale University, New Haven, CT 06520; ^b^Nanobiology Institute, School of Medicine, Yale University, West Haven, CT 06516; ^c^Laboratoire de Physique de l’Ecole normale supérieure, Département de Physique, Ecole Normale Supérieure, Université Paris Sciences & Lettres CNRS, Sorbonne Université, Université de Paris, Paris F-75005, France

**Keywords:** Munc13-1 clusters, neurotransmission, membrane fusion, synaptic vesicle, diacylglycerol

## Abstract

Munc13-1 is essential for vesicle docking and fusion at the active zone of synapses. Here, we report that Munc13-1 self-assembles into molecular clusters within diacylglycerol-rich microdomains present in phospholipid bilayers. Although the copy number of Munc13-1 molecules in these clusters has a broad distribution, a systematic Poisson analysis shows that this is most likely the result of two molecular species: monomers and mainly hexameric oligomers. Each oligomer is able to capture one vesicle independently. Hexamers have also been observed in crystals of Munc13-1 that form between opposed phospholipid bilayers [K. Grushin, R. V. Kalyana Sundaram, C. V. Sindelar, J. E. Rothman, *Proc. Natl. Acad. Sci. U.S.A.* **119**, e2121259119 (2022)]. Mutations targeting the contacts stabilizing the crystallographic hexagons also disrupt the isolated hexamers, suggesting they are identical. Additionally, these mutations also convert vesicle binding from a cooperative to progressive mode. Our study provides an independent approach showing that Munc13-1 can form mainly hexamers on lipid bilayers each capable of vesicle capture.

## Significance

Munc13-1 is a molecular chaperon that facilitates recruitment and docking of synaptic vesicles at the active zone of the synapse. Using model membranes and statistical modeling, we show that Munc13-1 forms mainly hexamers on diacylglycerol rich microdomains on lipid membranes. These hexamers act cooperatively to capture vesicles from a solution. Statistics show that each hexamer binds to one vesicle. Disruption of the interactions at Munc13-1 hexagonal interface by point mutations based on crystallographic data alters its oligomerization state and hexamers are no longer observed. Furthermore, the mutant oligomer loses its cooperativity in binding vesicles. Our study suggests that the Munc13-1 hexamers observed on lipid bilayers resemble the hexagons revealed by crystallography.

Many cellular proteins form oligomeric structures to achieve their specific biological functions (1–3). Numerous proteins required for neurotransmitter release are concentrated within the crowded active zones of presynaptic terminals, potentially favoring the assembly of well-defined oligomeric structures that would not be observed in dilute solution. Relatively small differences in energy among a series of alternative oligomeric arrangements could then enable successive rearrangements to occur in a defined order, thereby choreographing the process of vesicle capture and activation for fusion. In this paper, we will report properties of oligomers of the synaptic protein Munc13-1 which assemble on lipid bilayers following concentration within diacylglycerol (DAG)-rich microdomains likely mimicking portions of the presynaptic active zone.

Munc13-1 is a key chaperone protein required for the assembly of the SNARE complex of Syntaxin-1, VAMP2, and SNAP25 which ultimately triggers membrane fusion for synaptic transmission (4–14). Munc13-1 is a large molecule, the C-terminal half of which contains its characteristic MUN domain (residues 859–1531), flanked at one end by a DAG-binding C_1_ domain and a PIP_2_ and Ca^++^-binding C_2_B domain, and at the other end by its phosphatidyl-serine (PS)-binding C_2_C domain. Munc13-1 is initially required to capture synaptic vesicles via its C_2_C domain (15, 16); it is later required to activate Syntaxin1 to initiate SNAREpin assembly (17) during which it interacts with each of the three synaptic SNARE proteins (17–19), and approximately 5 to 10 copies remain in close association with each primed, ready-release vesicle (20, 21) most likely containing 6 clamped central SNAREpins arranged symmetrically on a ring (22, 23). Munc13-1 is therefore ideally positioned to choreograph this entire process via a series of oligomeric assemblies.

We previously reported that purified Munc13-1 protein reconstituted onto supported lipid bilayers containing DAG and PIP_2_ is organized into clusters containing from 2 to ~20 copies, as revealed by a combination of quantitative total internal reflection fluorescence (TIRF) microscopy and stepwise photobleaching (16). We noted that only clusters containing a minimum of 6 copies of Munc13-1 were capable of efficiently capturing and retaining PS-containing small unilamellar vesicles (SUVs). This hinted that a hexamer of Munc13 could be a basic functional unit for vesicle binding, a conclusion that had no structural precedent at the time.

Direct evidence for a hexameric organization then emerged when we solved the structure of two-dimensional crystals of Munc13-1 that spontaneously assemble between bilayers containing copious acidic phospholipids, revealing two oligomeric arrangements: a 21-nm high “upright” trimer that binds both bilayers and a 14-nm high “lateral” hexagon (24). The hexameric structure of Munc13-1 is particularly of interest because it may represent a fusion intermediate, with six Munc13-1-dependent protein complexes under each closely docked synaptic vesicle, which is consistent with the electron tomography studies which showed that the protein density between synaptic vesicles and the plasma membrane exhibited sixfold symmetry (22, 23).

The straightforward interpretation of these results (24) is that upright trimers first capture synaptic vesicles by PS-dependent binding to their plasma membrane-distal C_2_C domains and then transition closer to the plasma membrane as the trimers rearrange to form hexamers. However, this model would seem to require that the top surface of the hexamer retains the synaptic vesicles, even though the known vesicle binding surface in C_2_C is sterically unavailable in the hexamer (24). The structural model (24) and in vitro and in vivo functional assays (25, 26) also strongly predicted that DAG binding by the C_1_ domain should trigger the rearrangement of trimers into hexamers, which involves a simple intramolecular rigid body rotation of C_1_-MUN-C_2_C relative to C_2_B, which remains fixed on the plasma membrane surface (24). This followed because the DAG binding site is closely opposed to the bilayer surface in the hexamer but points away from the bilayer in the trimer.

In this study, we have built on the observation that clusters of approximately 6 copies of Munc13 bind a vesicle (16) to test these predictions. Specifically, we now investigate whether isolated hexamers of Munc13 can form on lipid bilayers, whether they require DAG to assemble, and whether each hexamer can potentially retain synaptic vesicles following their initial capture by upright trimers.

## Results and Discussion

### DAG Forms Microdomains within Phospholipid Bilayers.

**Fig. 1.**
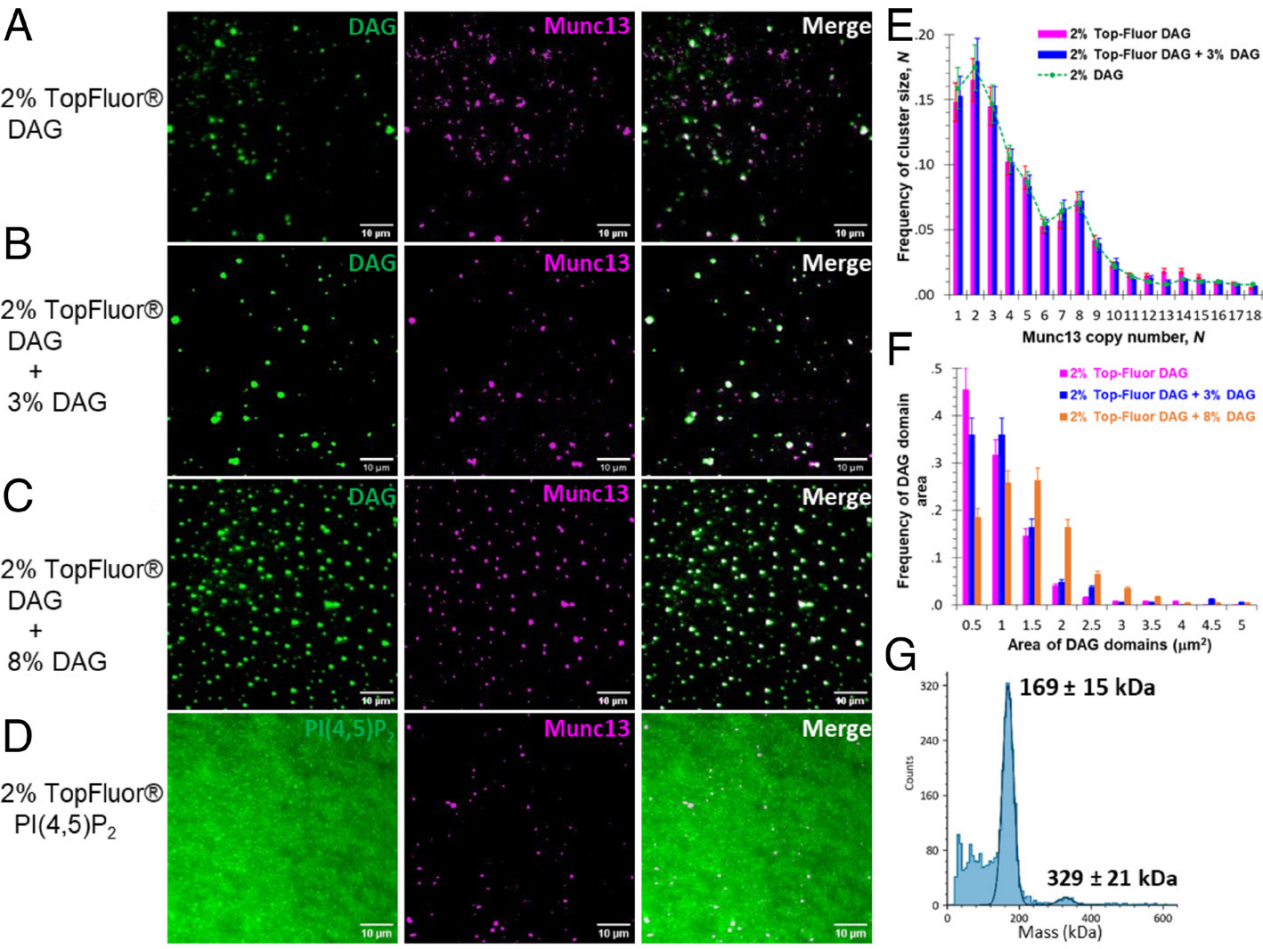
DAG forms microdomains on lipid bilayer, and wild-type Munc13-1 clusters are located at these microdomains. (*A*–*C*) Representative TIRF images of fluorescent DAG in bilayers and Munc13-1 bound to bilayers: (*A*) bilayer with 2% TopFluor® DAG, 0% nonfluorescent DAG, and 2% nonfluorescent PI(4,5)P_2_; (*B*) bilayer with 2% TopFluor® DAG, 3% nonfluorescent DAG, and 2% nonfluorescent PI(4,5)P_2_; and (*C*) bilayer with 2% TopFluor® DAG, 8% nonfluorescent DAG, and 2% nonfluorescent PI(4,5)P_2_. *Left* panels are fluorescent DAG, the middle panels are Munc13-1 labeled with Alexa 660, and *Right* panels are the merge of the previous two panels. (*D*) Representative TIRF images of fluorescent PI(4,5)P_2_ in bilayers which contains 2% nonfluorescent DAG and 2% TopFluor® PI(4,5)P_2_. The *Left* panel is fluorescent PI(4,5)P_2_, the *Middle* panel is Munc13-1 labeled with Alexa 660, and *Right* panels are the merge the previous two panels. (*E*) Distributions of the copy number of Munc13-1 molecules in the clusters with various DAG amounts (sample size: *n = 4* for 2% TopFluor DAG; *n = 3* for 2% TopFluor DAG + 3% DAG; and *n = 5* for 2% DAG); and (*F*) Distribution of the area of DAG microdomains (sample size: *n = 4* for 2% TopFluor DAG; *n = 3* for 2% TopFluor DAG + 3% DAG; and *n = 3* for 2% TopFluor DAG + 8% DAG). (*G*) The molecular weight of Munc13C-Halo-Alexa 660 in solution was measured with a Mass Photometer. Final concentration of Munc13C-Halo-Alexa 660 in this measurement was around 10 nM. The experimental molecular mass nearly equals its theoretical molecular weight.

DAG is a lipid in the plasma membrane at relatively low concentration. However, the membrane lipid phosphatidylinositol 4,5-bisphosphate [PI(4,5)P_2_] is known to form a clustered domain in lipid bilayers in the presence of the Syntaxin-1 juxtamembrane domain (27). Consequently, DAG may exist in the form of clustered microdomains when such enriched PI (4,5)P_2_ is hydrolyzed to DAG by the phospholipase C (28–30). DAG has been reported to induce phase transition in phospholipid bilayers (31). Molecular dynamics simulations suggest that DAG may form domains in the bilayers (32). Hence, such DAG domains may have been present in our previous study on Munc13-dependent vesicle binding (16). To test this directly, we examined the distribution of a fluorescently labelled analogue of DAG, TopFluor® DAG (33), replacing ordinary DAG (2 mol%) in bilayers also comprised of 71 mol% PC, 25% PS, and 2% PI(4,5)P_2_, the same composition used in our previous study (see *Materials and Methods* for acronyms). Fig. 1 *A* (*Left*) confirms that microdomains of DAG were indeed present. These DAG-rich domains ranged in area from less than 0.5 μm^2^ up to about 2 μm^2^, averaging 0.7 ± 0.5 μm^2^ for 2 mol% total DAG (Fig. 1*F*, red bars). Higher concentrations of DAG tested up to a total of 10 mol% total (2 mol% TopFluor® DAG analogue with the balance of DAG being underivatized DAG) resulted in somewhat larger microdomains, the largest being 2 to 3 μm^2^ at 10% total DAG (Fig. 1*F*). The average DAG domain was 0.8 ± 0.7 μm^2^ at 5 mol% total DAG and 1.2 ± 0.8 μm^2^ at 10 mol% total DAG.

### Munc13-1 Binds Preferentially to the DAG Microdomains.

We next tested whether Munc13-1, previously shown (16) to bind to DAG-containing bilayers in clustered regions that could not be resolved optically, might be selectively localized within DAG domains. For this purpose, we employed a minimal fragment of Munc13-1 known to be functional in vivo (9, 19), consisting of C_1_-C_2_B-MUN-C_2_C (residues 529 to 1,735; Munc13C) with a Halo domain added at its C-terminus (Munc13C-Halo). This protein was rendered fluorescent by reacting the Halo tag with an Alexa Fluor 660 ligand. The theoretical molecular weight of this protein is 167 kDa. It is soluble in buffer solution. In mass photometry measurements, it displayed a large peak at 169 ± 15 kDa and a barely noticeable second peak at 329 ± 21 kDa (Fig. 1*G*). This confirmed that the starting material in our experiments, 10 nM Munc13-1 in solution, was primarily momomeric with an extremely small fraction of dimers.

We incubated the supported lipid bilayer with such 10 nM Munc13-1 solution. Then, we simultaneously imaged DAG (TopFluor®, 488 nm) and Munc13C (Alexa 660) on the lipid bilayer by TIRF.

Fig. 1 *A*–*C* (*Middle*) reproduces our original finding that Munc13C forms clusters and goes on to present additional data that establish that at 2 to 10 mol% DAG almost all of these Munc13-1 clusters are within the DAG microdomains (Fig. 1 *A*–*C*, panels at *Right*). This suggests that DAG locally increases its own concentration through self-aggregation, resulting in DAG microdomains which recruit and locally concentrate Munc13C molecules.

The number of copies of Munc13C in each DAG domain was then determined by a combination of quantifying the TIRF intensity and stepwise photobleaching as before [(16), *Material and Methods*]. The frequency distributions of copy numbers were indistinguishable at 2 mol% (red) and 5 mol% (blue) DAG, and the same whether the DAG was fluorescently tagged (red) or natural DAG (dashed line), showing that Munc13-1 binding is not affected by the fluorescent probe. Very little binding or clustering of Munc13-1 occurred when DAG was omitted from the bilayers (*SI Appendix*, Fig. S1).

Because Munc13-1 can potentially bind to the plasma membrane either to PI(4,5)P_2_ (via C_2_B) or to DAG (via C_1_) or to both lipids simultaneously, it was of special interest to determine whether PI(4,5)P_2_ is excluded from the DAG microdomains. For this purpose, we prepared bilayers containing 2 mol% nonfluorescent DAG and 2 mol% TopFluor® PI(4,5)P_2_. We observed that PI(4,5)P_2_ was nearly uniformly distributed in the lipid bilayer, and Munc13-1 clusters were formed in the PI(4,5)P_2_ region (Fig. 1*D*), which suggested that PI(4,5)P_2_ was neither concentrated within nor excluded from the DAG microdomains. This implies that Munc13-1 bound to DAG microdomains can potentially be bound to both DAG and PI(4,5)P_2_ simultaneously.

Mass photometry experiments showed that Munc13-1 remained as monomer in solution, from 8 nM to 170 nM, (*SI Appendix*, Fig. S2). This result indicates that DAG, in addition to locally increasing the Munc13-1 concentration, also provides a binding interface for correctly orientating Munc13-1 for oligomer formation, as already suggested by the previous cryoelectron tomography study.

### Poisson Distribution Analysis Reveals That Munc13-1 Assembles into Discrete Oligomers within DAG Microdomains, Most Likely Hexamers.

As reported previously (16), we are able to determine the number of Munc13-1 molecules, *N*, in each cluster. For a given cluster, in principle, such *N* Munc13-1 molecules may exist in the form of monomers or oligomers, or a combination of these states. The likelihood of lateral diffusion of the Munc13-1 molecules within the clusters makes it difficult to determine their oligomeric state from optical microscopy alone, and this difficulty is compounded when the domains are close in size to the wavelength of the light. For example, a “cluster” with a copy number of 6 Munc13-1 molecules (*N* = 6) could be at one extreme 6 individual, noninteracting monomers diffusing freely over the surface of a common lipid microdomain, or at the other extreme a rigidly structured hexamer such as is observed bound to bilayers in the Munc13-1 crystal (24).

**Fig. 2.**
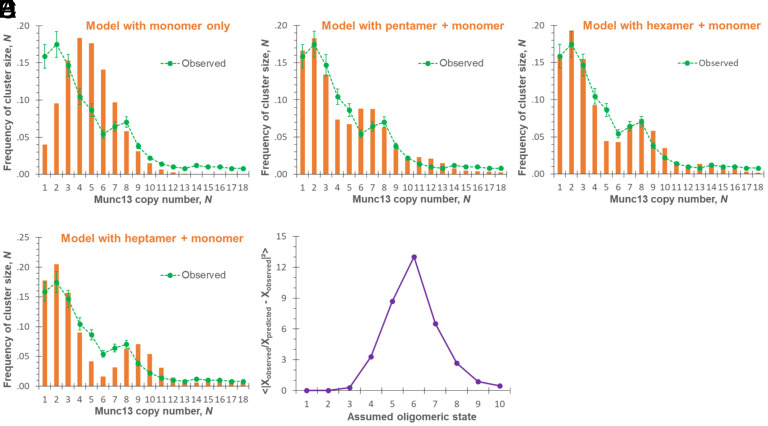
Poisson distribution modeling of oligomers in wild-type Munc13-1 clusters. (*A*) Single Poisson distribution, assuming all wild-type Munc13-1 in monomer state; (*B*) through (*D*) Dual Poisson distribution (monomer + uniform size oligomer), 2-step process: monomer with pentamer (*B*), monomer with hexamer (*C*), and monomer with heptamer (*D*). The orange bars represent predicted distribution of the copy number of Munc13-1 molecules in the clusters, and the green dashed lines are experimental data (sample size *n = 5*). (*E*) The *O* value of the Poisson modelling as function of the assumed oligomeric state of wild-type Munc13-1. Larger *O* value suggests that the predicted oligomeric state fits better with observed data.

To rule out the trivial explanation (null hypothesis) that the observed Munc13-1 clusters are merely a collection of individual proteins that happen to be bound to the same lipid microdomain, we can employ well-understood Poisson statistics to analyze the copy number distribution of the apparent Munc13-1 clusters. In the null hypothesis, the frequency distribution of copy numbers will be given by:

[1] $$P_{M}\left(m\right)=\frac{\langle {m\rangle }^{m}}{m!}e^{-\langle m\rangle },$$

where m   is the number of monomers and 〉*m*〈 the mean copy number of Munc13-1 molecules per cluster. 〉*m*〈 cannot be directly measured experimentally since the domains without any Munc13-1, i.e., $P_{M}\left(0\right)$   , is unknown because in most experiments, we used unlabeled DAG. Hence, the observed copy number probability needs to be corrected for it. This is a straightforward process (*SI Appendix*, *Supporting Text*). This model assumes that the DAG microdomains are of uniform size which is reasonable considering the measured area distribution. With these considerations, we determined that 〉*m*〈 ≈ 4.8 copies of Munc13-1 per cluster at 2 mol% total DAG. This predicts a copy number distribution (Fig. 2*A*, bars) that clearly does not match with the observed distribution (dashed line). We can conclude that the null hypothesis is incorrect. That is, the observed copy number distribution can only be explained by molecular interaction among the bound Munc13-1 into stable oligomers which distribute as such among the individual cluster.

We can further refine the statistical model to provide deeper insight into the degree of oligomerization of Munc13-1 that is likely involved, which we term *“K*”. For example, if monomers were to assemble exclusively into hexamers, then *K* = 6, and a cluster containing 8 copies of Munc13-1 (*N* = 8) would in fact most likely contain 1 copy of a hexamer and 2 unassembled monomers or, less likely, 8 copies of individual Munc13-1. More generally, we will determine the best fit for *K* to the experimental data with the assumption that Munc13-1 is present in clusters only as combinations of monomers and *K*-mers. These two populations behave independently of each other and each randomly distributes among clusters following its own Poisson probability distribution, exactly as in the first model:

[2] $$\begin{array}{c} P_{M}\left(m\right)=\frac{\langle {m\rangle }^{m}}{m!}e^{-\langle m\rangle }\\ P_{K}\left(k\right)=\frac{\langle {k\rangle }^{k}}{k!}e^{-\langle k\rangle } \end{array},$$

where *P_M_* and *P_K_* are the probability distributions for monomers and *K*-mers, respectively, and *m* and *k* are respectively the numbers of copies of monomers and *K*-mers in the cluster.

The observed copy number distributions of Munc13 cluster result from combining the monomers and *K*-mer distributions, as presented in *SI Appendix*, Table S1. 〉*m*〈 and 〉*k*〈 can then be calculated (see *SI Appendix*, *Supporting Text* for explanations). The predicted probability distribution of copy numbers is fully described by these two parameters (*SI Appendix*, Eq. **S5**).

The resulting copy number distributions predicted from dimer to decamer, *K* = 2 to *K* = 10, are shown in Fig. 2 *B*–*D* and *SI Appendix*, Fig. S3. The predicted distribution for hexamers (*K* = 6) closely matches the experimental results (dashed lines). In particular, this model correctly predicts the second peak at *N* = 8 and the third peak at *N* = 14. Flanking models (*K* = 5 and *K* = 7) do not predict these observed peaks. To objectively assess which model(s) seems to describe best the observed cluster size distribution, we use a parameter to test the oligomerization degree, *O*, that quantitatively compares the predicted and observed histograms for each *K*-mer. A larger *O* value indicates a better prediction of the experimental distribution. The results confirm that the hexamer model has the highest *O* value (Fig. 2*E* and *SI Appendix*, *Supporting Text*).

Finally, another prediction from this model is that the fraction of DAG domains without any Munc13-1 is:

[3] $$P_{\mathrm{Total}}\left(0\right)=e^{-\langle k\rangle }e^{-\langle m\rangle }.$$

In the case of hexamers, 〉*m*〈 = 2.4 and 〉*k*〈 = 0.375, which leads to $P_{\mathrm{Total}}\left(0\right)=0.06=6\%$ . Experimentally, we can estimate $P_{\mathrm{Total}}\left(0\right)$ from experiments with bilayers containing fluorescent DAG. We find 9 ± 2% of DAG domains lack Munc13C, which is reasonably close to the predicted value considering the simplicity of our model.

### Targeted Mutations Predicted to Destabilize the Hexamers Observed in Crystals Alter the Munc13-1 Copy Number Distribution and Disrupts Co-Operative Vesicle Binding.

**Fig. 3.**
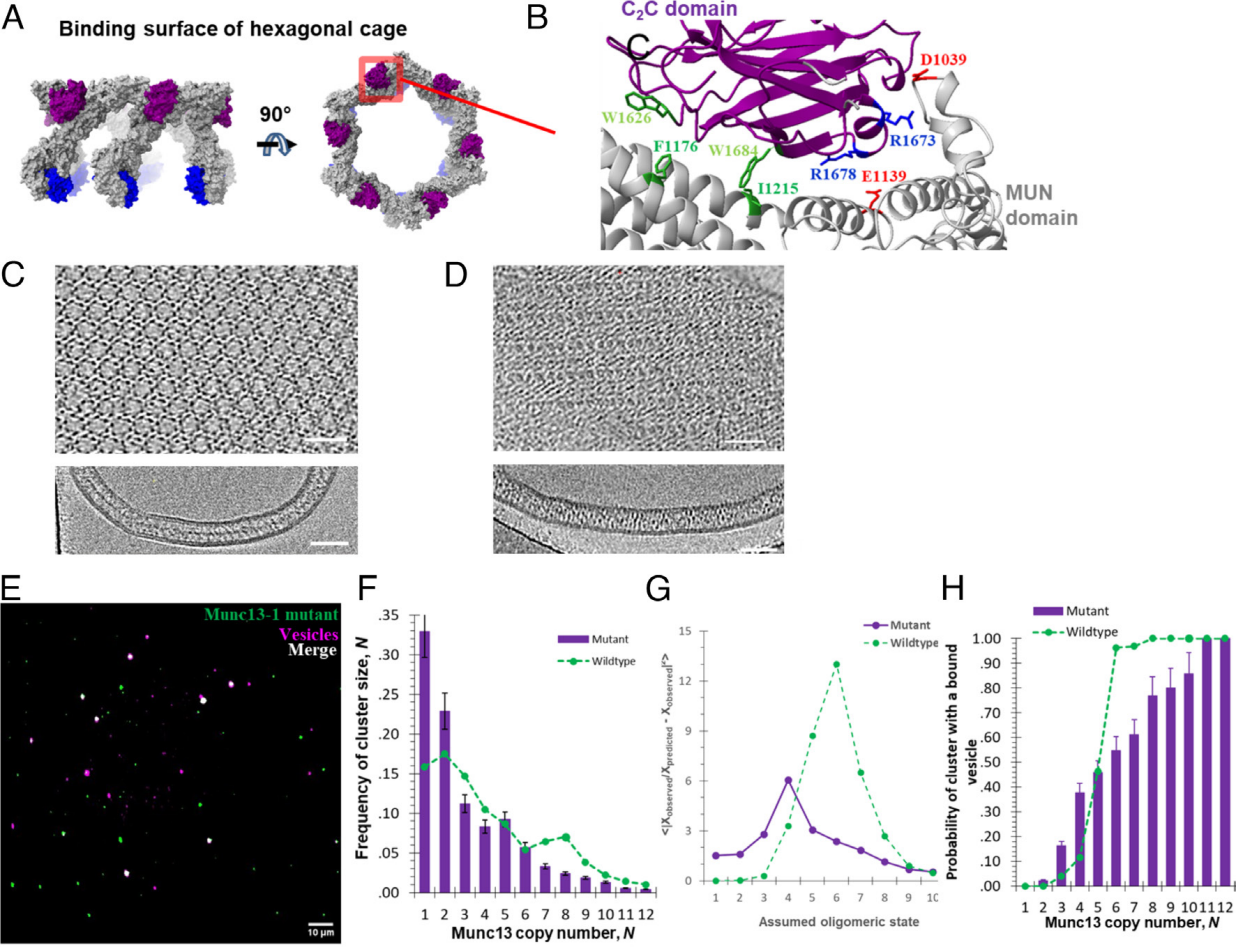
Mutations in interaction interface between C_2_C and neighboring MUN domain in the Munc13-1 hexagon affect the protein organization, cluster size distribution, and vesicle capture. (*A*) Top and side views of the hexagonal cage of Munc13-1 oligomer that binds a vesicle (based on PDB 7T7C). C_2_C domains are colored in purple, MUN domains in gray, and C_2_B in blue. The red rectangular area shows the position of interaction interface between C_2_C and MUN domain in Munc13-1 hexagon. (*B*) Close-up view of interface in ribbon representation. Possibly involved residues in interaction are labeled. The interface contains two separate regions: hydrophobic (green-colored amino acid residues) and polar (red and blue–colored amino acid residues). (*C*) and (*D*) Slices through the reconstructed tomograms. (*C*) Wild-type Munc13C hexagonal crystal between negatively charged lipid bilayers. The *Top* panel is the Topaz-denoised (34) top view of the crystal. Bottom is the side view of the crystal with visible protein density lane in the middle between lipid bilayers corresponding to the top of hexagons (*A*) formed by lateral conformation of Munc13-1. (*D*) The *Top* panel is the Topaz-denoised top view of the crystal formed by F1176N/I1215N/E1139R/D1039R Munc13-1 mutant. This crystal is characterized as a rectangular lattice with denser protein packing in the form of rows of round-shaped densities and protein-filled space between them. The *Lower* panel is the slice through the side of the crystal. The middle protein density lane is no longer visible. (Scale bars, 50 nm.) (*E*) Overlay of TIRF image of Munc13-1 interface mutant labeled with Alexa 488 on a lipid bilayer membrane containing PC, PS, DAG, and PI(4,5)P_2_, with TIRF image of captured vesicles by the same Munc13-1 interface mutant (vesicles labeled with Atto647N, pink). (*F*) Distribution of the copy number of Munc13-1 interface mutant molecules in the clusters (purple columns) (sample size *n* = *4*). The green dots represent the distribution of the copy number in the clusters formed by wild-type Munc13-1, which serves as a reference. (*G*) The *O* value of the Poisson modelling as function of the assumed oligomeric state of the Munc13-1 interface mutant (purple solid line). The green dashed line represents the *O* value of wild-type Munc13-1 clusters, which serves as a reference (*H*) Probability of vesicle capture by the clusters of Munc13-1 interface mutant (purple columns) as a function of their cluster size which is the copy number of the protein molecules in the cluster (sample size *n* = *4*). The green dashed line represents the probability of wild-type Munc13-1 clusters, which serves as a reference.

Are the predominantly hexameric units of Munc13-1 that assemble in clusters on the surface of single lipid bilayers structurally equivalent to the lateral hexagons that assemble in protein crystals between bilayers (24)? The lateral hexamer assembles when the Munc13-1 molecules are in their “closed” conformation and when the C_2_C domain of one copy contacts the MUN domain of its neighbor. This geometry results in a closed hexameric ring (Fig. 3*A*).

It was previously shown by cryoelectron tomography that Munc13-1 forms a hexagonal lattice between negatively charged lipid bilayers (24). We located possible interacting amino acids within the lateral interfaces between the C_2_C domain and MUN domain of the neighbor molecule in the hexagonal formation of Munc13-1 (Fig. 3*A*). This interface was proposed to consist of two regions—hydrophobic (a/a F1176 and I1215 on MUN domain and corresponding interaction partners W1626 and W1684 on C_2_C domain) and polar (a/a E1139 and D1039 on MUN domain and R1678, R1673 on C_2_C domain) (Fig. 3*B*). These two regions are well conserved in vertebrates, and the hydrophobic region is conserved in invertebrates as well (*SI Appendix*, Fig. S4).

To test selected amino acid residues, we mutated them on the MUN domain to N1176, N1215, R1139, and R1039. We refrained from mutating the corresponding residues on the C_2_C domain due to possible negative effect on membrane binding abilities. This quadruple mutation not only disrupted the formation of the hexagonal crystal but also led to the assembly of a rectangular crystal lattice (Fig. 3*D* and *SI Appendix*, Fig. S5). Visually protein packing is much denser than wild type with visible rows of round-shaped protein densities and diagonally placed protein connections between them (Fig. 3 *C* and *D*). When measured between the centers of round elements, rows are spaced by 35 nm, and round elements within a row are spaced by 12 nm. Distance between bilayers as measured on the side views (Fig. 3 *C* and *D*) is similar and ~21 nm suggesting the presence of upright open configuration of the protein in the crystal. The 3D reconstruction of a new crystal is ongoing and will hopefully show the details of a new crystal assembly.

If the oligomers assembling on Munc13-1 clusters on lipid bilayers are in fact lateral hexagons in the cryoelectron tomography study, they too should be disrupted by these mutations. To test this, we used the same mutant version of Munc13C as above, F1176N, I1215N, E1139R, and D1039R and measured its copy number distribution and vesicle capture property (Fig. 3*E*). The mutant bound to DAG-containing lipid bilayers and formed cluster, similar to the wild-type protein, but had a markedly reduced copy number distribution (Fig. 3*F*). In particular, the predicted peaks for hexamers at *N* = 8 and *N* = 14 are abolished. Instead, a new peak is observed at *N* = 5. Applying the same model and methodology as for the Poisson analysis of the wild-type protein (Eq. **2** and *SI Appendix*, Eq. **S5**) reveals that only a tetramer (*K* = 4) model predicts the new peak at N = 5 (*SI Appendix*, Fig. S6), and that a tetramer has by far the largest *O* value (Fig. 3*G*). Further work involving direct structure determination will be required to independently confirm this conclusion.

In any case, the results here imply that C_2_C is required for the observed hexameric clustering of Munc13C. In our previous report, we suggested that C_2_C could be deleted without affecting clustering (Fig. 4*B* in ref. 16). With the perspective of the current work, re-examination reveals that clustering without C_2_C results in smaller clusters and the distribution notably differs from that of wild-type especially at copy numbers of 6 and 12, suggesting that there is a C_2_C-independent nonhexameric form of clustering that can occur when C_2_C is artificially removed.

**Fig. 4.**
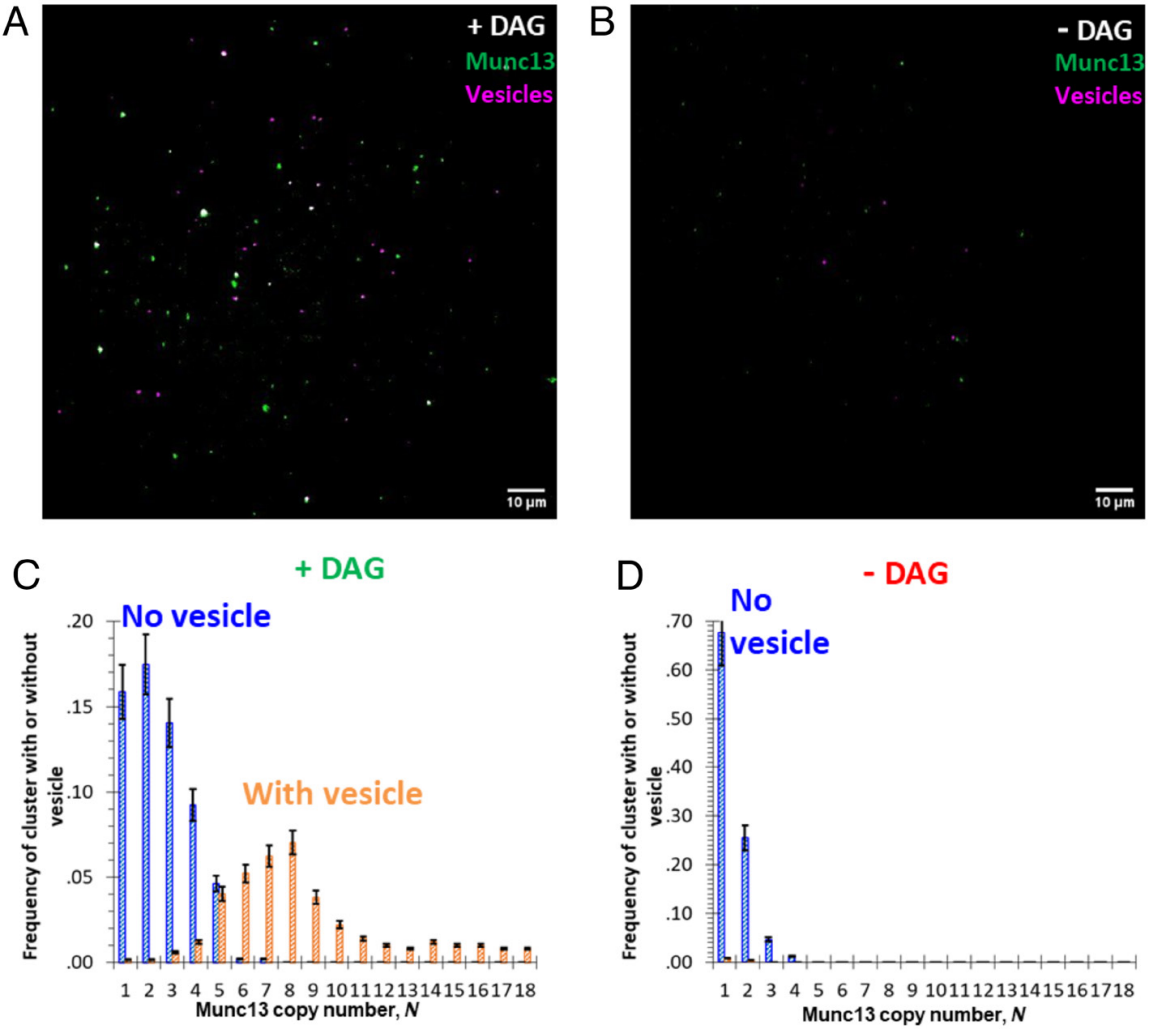
DAG plays a critical role in wild-type Munc13-1’s function of vesicle capture. (*A*) Overlay of TIRF image of wild-type Munc13-1 clusters formed on a bilayer with 2% DAG (wild-type Munc13-1 labeled with Alexa488, green) with the TIRF image of captured vesicles (vesicles labeled with Atto647N, pink). (*B*) Overlay of the TIRF image of wild-type Munc13-1 clusters that were formed on a bilayer in the absence of any DAG (Munc13-1, green) with vesicles bound to the same bilayer (vesicles, pink). (*C*) Distribution of the copy number of wild-type Munc13-1 molecules in the clusters on lipid bilayer in the presence of DAG that are capable (orange) and incapable (blue) of capturing vesicle, respectively (sample size n = 5). (*D*) Distribution of the copy number of wild-type Munc13-1 molecules in the clusters on lipid bilayer in the absence of DAG that are capable (orange) and incapable (blue) of capturing vesicle, respectively (sample size n = 4).

We previously reported that Munc13-1 clusters formed on DAG-containing lipid bilayers bound PS-containing SUVs depending on the number of copies of Munc13-1 present (16). In particular, we found that clusters containing 6 or more copies of Munc13-1 always captured a vesicle, and those with 4 or fewer copies almost never did, suggesting that 6 copies of Munc13-1 optimally co-operate to capture a vesicle when Munc13-1 was bound to DAG via its C_1_ domain, as disrupting the interaction between DAG and C_1_ reduced recruitment of Munc13-1 (*SI Appendix*, Fig. S7), while PI(4,5)_2_ has a partial contribution to the recruitment but little effect on the size distribution of the clusters (*SI Appendix*, Fig. S8). We have confirmed and extended these data in Fig. 4, which now tests the important prediction that vesicle binding, relying as it does, on Munc13-1 binding should likewise be DAG-dependent (compare Fig. 4 *A* and *B*, with and without DAG, respectively). Comparing the Munc13-1 copy number content of those clusters which fail to capture a vesicle (Fig. 4*C*, blue bars) with those that do (orange bars) confirms that it is only when the cluster contains 6 or more copies that it does not fail to capture a vesicle. This is also true for Munc13-1 clusters on the bilayer in the absence of DAG, where there is barely any colocalization with captured vesicles because the copy numbers of Munc13-1 molecules in these clusters are usually less than 6 (Fig. 4*D*).

These experiments cannot in and of themselves distinguish whether the 6 co-operating copies of Munc13-1 are preassembled into a hexamer as distinct from assembling around a vesicle during the process of capturing it. However, the former model makes a unique prediction, that disrupting the hexamers should eliminate the co-operativity of vesicle binding. To test this, fluorescent vesicles containing 68 mol% PC, 30% PS, and 2% PE-Atto647N were incubated with supported lipid bilayers harboring the Munc13-1 quadruple mutant (F1176N I1215N E1139R and D1039R) clusters. The bilayers were then washed to remove unbound vesicles and imaged using TIRF microscopy (Fig. 3*E*). The results were quantified by measuring the number of copies of the Munc13-1 mutant in each cluster and scoring that cluster for the presence or absence of vesicles (Fig. 3*H*). As predicted for co-operative vesicle binding to hexamers, the hexamer-destabilizing mutations (purple bars) reduced the probability of vesicle capture at *N* = 6, the threshold observed for co-operative binding by the wild-type protein (Fig. 3*H*, green dashed line, reproduced from ref. 16).

As strikingly, co-operativity was eliminated. Vesicle capture by the mutant increases progressively (nearly linearly) with the number of copies present. This suggests that each copy of the mutant, unable to form hexamers, now binds to vesicles independently.

### Each Hexamer Appears to Capture a Single Vesicle.

**Fig. 5.**
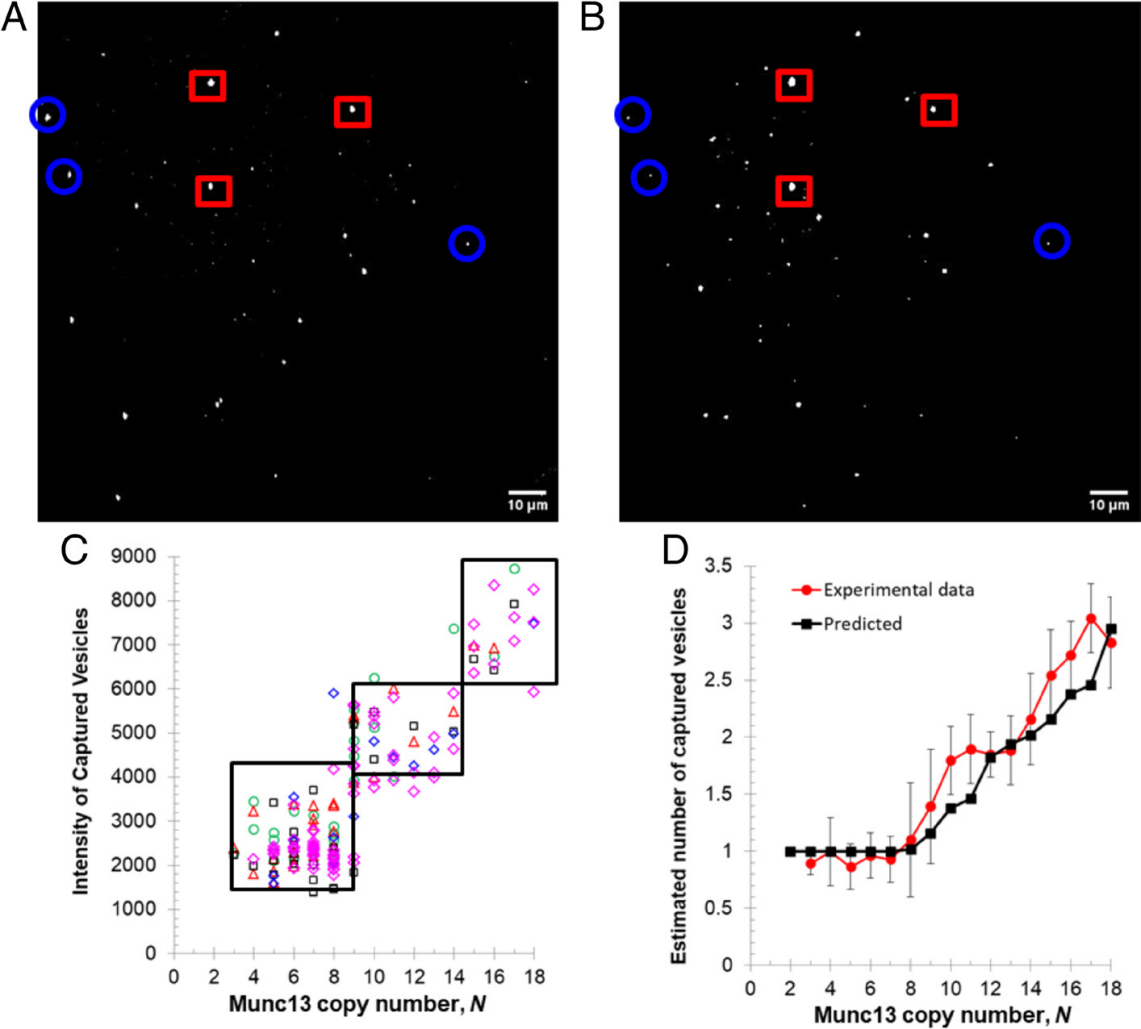
Quantal relationship of the number of captured vesicles by a cluster and the number of copies of Munc13-1 molecules in the same cluster. (*A*) and (*B*) Qualitative correlation between optical cluster sizes and the optical sizes of captured vesicles. When the intensities of Munc13-1 clusters (*A*) are bigger, the corresponding intensities of captured vesicles (*B*) are larger. (*C*) Intensity distribution of captured vesicles as a function of the copy numbers of Munc13-1 molecules in the corresponding clusters shows three distinct regimes. Different symbols and colors represent the data points were from different independent experiments, sample size *n = 5*. (*D*) Comparison of the predicted number of captured vesicles as a function of the copy number of Munc13-1 molecules in the cluster (black squares), and experimental average number of captured vesicles that was determined from vesicle intensities, versus the copy number of Munc13-1 molecules in the cluster (red spheres).

So far, we have not attempted to distinguish the number of vesicles bound to each Munc13-1 cluster. Two vesicles bound to the same cluster would be difficult or impossible to resolve optically. However, the fluorescent intensities of these vesicles would be additive. With this in mind, we measured the total vesicle fluorescence intensity colocalizing with each Munc13-1 cluster and related it to the number of copies of Munc13-1 bound to the same cluster (Fig. 5 *A* and *B*). There is a clear increase in vesicle intensity with the copy number of Munc13-1 molecules in the cluster (Fig. 5*C*).

The simplest model would be that each hexamer can bind one vesicle. If this were the case, we would observe a quantal rather than a progressive increase in the number of vesicles bound as a function of copy number. In particular, these steps should occur around *N* > 8 and *N* > 14, where one and two hexamers predominate (Fig. 2*C*). In fact, this is exactly what we observe (Fig. 5*C*).

Because the size of each vesicle (and thus its intensity) follows a probability distribution, it is not necessarily straightforward to relate the intensities in each step to the number of vesicles, even though the vesicles in the capture experiments have a narrow size distribution with a mean radius of ~31 ± 9 nm (*SI Appendix*, Fig. S9). However, this can be done with a normalization process that takes into account the various combinations of monomers and hexamers that can combinatorially result in a given copy number for a cluster (*SI Appendix*, Table S1) and their associated probabilities for vesicle binding. As a result, we can calculate the mean number of vesicles bound to a cluster as a function of its overall copy number as predicted by the quantal binding model (Fig. 5*D*). The good agreement between the predictions and observations suggests that our assumptions are correct and that each DAG-dependent hexamer captures a single vesicle as an independent co-operative unit.

## Concluding Remarks

In summary, a combination of biochemical and structure-based mutation data implies that hexamers corresponding to the lateral hexamers in protein-bilayer crystals (24) form as stand-alone structures on DAG-rich microdomains (Figs. 1–3); that hexamer assembly depends on binding to DAG microdomains (*SI Appendix*, Fig. S1*C*); and that each hexamer co-operatively captures a single vesicle (Fig. 5). Therefore, hexamers are predicted to bind two bilayers simultaneously. It will be important to directly visualize such structures by cryoelectron microscopy in future studies.

Our conclusion that hexamers can still bind vesicles is satisfying biologically because it would explain how a synaptic vesicle captured initially by the C_2_C domains of upright trimers of Munc13-1 (21 nm high) could be retained at the plasma membrane after these trimers have putatively transitioned into hexamers (now 14 nm high) as Munc13-1 transitions from its open to closed conformation, putatively driven by DAG binding. Yet, this conclusion is also surprising from a structural perspective. The upright trimers bind bilayers representing the vesicle by their C_2_C domains, and our cryoEM structure (24) directly visualized the surface of C_2_C that would be in contact with a synaptic vesicle. But as we have detailed (24) in the closed conformation of the subunits of a hexamer, this very surface of C_2_C is engaged in binding to the neighboring MUN domain and thus sterically unavailable.

Presumably a new surface is created on the top of the hexamer that contains a combination of hydrophobic and basic residues which can multivalently bind negatively charged PS-containing phospholipid vesicles (16). It remains for future work to identify the constituents of this unanticipated vesicle-binding surface.

Moreover, our work suggest that oligomerization of synaptic proteins is a critical form of organization at the presynaptic membrane. Many important tethers in synaptic fusion, such as the RIM protein, RIM-BP, and Synapsin, are known to undergo phase separation and form condensed liquid droplets in the active zone (35, 36). These droplets are a form of oligomerization. These findings show that oligomerization occurs quite often and is probably a general mechanism for locally obtaining high concentration.

## Materials and Methods

### Chemicals.

4-(2-Hydroxyethyl)piperazine-1-ethanesulfonic acid (HEPES), Potassium hydroxide, Potassium chloride (KCl), Magnesium chloride (MgCl_2_), Glycerol, DNAse I, RNAse A, Benzonase, Roche complete protease inhibitor cocktail tablets, Phorbol 12-myristate 13-acetate, and DL-Dithiothreitol (DTT) were purchased from Sigma-Aldrich. Nickel-NTA agarose, TCEP-HCl, and Expi293™ Expression System Kit were supplied by Thermo Fisher Scientific. The lipids used in this study, 1,2-dioleoyl-sn-glycero-3-phosphocholine (DOPC), 1-palmitoyl-2-oleoyl-sn-glycero-3-phosphocholine, 1,2-dioleoyl-sn-glycero-3-(phospho-L-serine) (sodium salt) (DOPS), L-α-phosphatidylinositol-4,5-bisphosphate (Brain, Porcine) (ammonium salt) [brain PI(4,5)P_2_], 1-2-dioleoyl-sn-glycerol (DAG), 1,2-dioleoyl-sn-glycero-3-phosphoethanolamine-N-(7-nitro-2-1,3-benzoxadiazol-4-yl) (ammonium salt) (DOPE-NBD), 1-palmitoyl-2-(dipyrrometheneboron difluoride)undecanoyl-sn-glycerol (TopFluor® DAG), 1-oleoyl-2-{6-[4-(dipyrrometheneboron difluoride)butanoyl]amino}hexanoyl-sn-glycero-3-phosphoinositol-4,5-bisphosphate (ammonium salt) [TopFluor® PI(4,5)P_2_], and 1-oleoyl-2-(6-((4,4-difluoro-1,3-dimethyl-5-(4-methoxyphenyl)-4-bora-3a,4a-diaza-s-indacene-2-propionyl)amino)hexanoyl)-sn-glycero-3-phosphoinositol-4.5-bisphosphate (ammonium salt) (TopFluor® TMR PI(4,5)P_2_), were purchased from Avanti Polar Lipids. 1,2-Dioleoyl-sn-gylcero-3-phosphoethanolamine ATTO 647N (DOPE-Atto647N) was from ATTO-Tec. The plasmid maxi prep kit was from QIAGEN. HaloTag® Alexa Fluor® 488 Ligand and HaloTag® Alexa Fluor® 660 Ligand were purchased from Promega. All aqueous solutions were prepared using 18.2 MΩ ultra-pure water (purified with the Millipore MilliQ system).

### Protein Constructs, Expression, and Purification.

The original vector expressing rat Munc13 was a kind gift from Claudio Giraudo. Similar to our previous report (16), the expression plasmids His_12__PreScission _C_1__C_2_B_MUN_C_2_C_tev_Halo and His_12__PreScission _C_1__C_2_B_MUN_C_2_C_tev_Halo quadruple mutant (F1176N I1215N E1139R D1039R) were produced by cloning rat Munc13-1 residues 529 to 1,735, respectively, to a pCMV-AN6 plasmid. Munc13-1 residues 1,408 to 1,452 were deleted and residues EF were added in their place (37). A short linker sequence containing a TEV cut site was subcloned in followed by the Halo tag. For CryoET studies, the cDNA construct with His_12_–Munc13C (Munc13-1 residues 529 to 1,735 with residues 1,408 to 1,452 replaced by the sequence EF) was expressed in a modified pCMV–AN6 vector, which includes a PreScission cut site following the His12 tag. The quadruple mutant (F1176N/I1215N/E1139R/D1039R) was generated using a QuickChange mutagenesis kit (Agilent Technologies).

The resulting plasmids were amplified with maxi prep using the QIAGEN Plasmid Maxi kit and were used to transfect Expi293F™ human cells. Proteins were expressed with the Expi293™ expression system following the manufacturer’s protocol.

The Munc13-1 proteins were then purified using Ni-NTA affinity beads as described before (38–40). To summarize, the cell pellet was thawed on ice and disrupted with a homogenizer and then spun in an ultracentrifuge for 30 min at ~142,400 × g at 4 °C. The supernatant was removed and 2 mL Qiagen Ni-NTA slurry along with 10 μL Benzonase were added and subsequently rotated using an orbiting wheel overnight at 4 °C. The beads were washed at 4 °C with 30 mL buffer containing 50 mM HEPES at pH 7.4, 400 mM KCl, 10% glycerol, 1 mM TCEP, and 10 mM Imidazole, then with another 30 mL buffer containing 50 mM HEPES at pH 7.4, 400 mM KCl, 10% glycerol, 1 mM TCEP, and 25 mM imidazole, followed the third 30 mL buffer containing 50 mM HEPES at pH 7.4, 270 mM KCl, 10% glycerol, 1 mM TCEP, and 25 mM imidazole. Then, 100 μL of PreScission protease (~2 mg mL^−1^) in 1 mL buffer was added to the beads and incubated for 3 h at room temperature with shaking to remove the 12xHis tag. After the cleavage reaction, elusions were collected and gel filtrated using a Superdex 200 column. The protein concentration was typically 1 to 2 mg mL^−1^ as determined by using a Bradford protein assay with bovine serum albumin as the standard.

### Protein Labeling.

The Munc13-1-Halo proteins were labeled by incubating the proteins with Alexa488 or Alexa660 conjugated with a Halo ligand from Promega, as described before (12). The protein was first centrifuged at 14,000 rpm for 20 min at 4 °C to remove any precipitation. Fluorescence dye was added into the protein solution at dye:protein = 5:1 molar ratio and the mixture was incubated for 30 min at room temperature with gentle rotation. Unreacted dye was removed by passing through the PD MidiTrap G-25 column (GE Healthcare) three times at room temperature. The labeling efficiencies were about 97%.

### Liposome Formation.

Protein-free liposomes were prepared by extrusion using an Avestin mini-extruder (6, 16). To make the liposomes for preparing bilayers, DOPC, DOPS, DAG, TopFluor® DAG, PI(4,5)P_2_, TopFluor® PI(4,5)P_2_, and/or TopFluor® TMR PI(4,5)P_2_ were mixed at proper mole ratio. Total amount was 3 μmoles. Nitrogen flow was used to remove the liquid solvents, and lipids were then dried in vacuum for 2 h, followed by resuspension with 500 μL buffer containing 50 mM HEPES, 140 mM KCl, and 10% glycerol. The resuspended lipids were treated with freezing (using liquid nitrogen) and thawing (in 37 °C water bath) cycles for 8 times, followed by extrusion using 100-nm or 50-nm membrane for 21 times.

We usually had 2% DAG, a reasonable estimate of the physiological DAG level, because it was previously reported that the DAG level is around 0.4% to 2% in hepatocytes (41). At the plasma membrane, PI(4,5)P_2_ is about 0.4 mol% to 4 mol% (42) and can be converted to DAG through hydrolysis (28). Both PI(4,5)P_2_ and DAG are able to be enriched to high local concentration in the presence of Syntaxin-1 juxtamembrane domain (27).

To make the liposomes for preparing vesicles, 68 mol% DOPC, 30 mol% DOPS, and 2 mol% DOPE-Atto647N were mixed. Total amount was 3 μmoles, and liposomes were produced similarly as above.

### Bilayer Preparation and TIRF Microscopy.

Bilayers were prepared by bursting liposomes on the glass surface using a glass-bottomed μ-Slide V1^0.5^ chip from Ibidi. Also, 2.5 µL MgCl_2_ at 500 mM was added into 122.5 µL buffer containing 50 mM HEPES (pH 7.4), 140 mM KCl, and 10% glycerol. Then, 125 µL extruded bilayer liposomes were added. Next, 60 µL MgCl_2_-liposome solution was loaded into the channel of the ibidi chip and incubated for 40 min at room temperature. The channel was washed with the same buffer supplemented with 6 mM EDTA and then with buffer supplemented with 1 mM DTT. Depending on the purpose of experiments, MgCl_2_ or CaCl_2_ may be added in the buffer to the desired concentration. Then, 60 µL of 10 nM Munc13-1-Halo-Alexa488 were loaded into the channel and incubate with the bilayer for 60 min at room temperature. The channel was washed with the buffer supplemented with 1 mM DTT. The vesicle liposomes were diluted 30 times. Subsequently, 60 µL diluted vesicle liposomes were loaded into the channel and incubated for 5 min at room temperature. The channel was washed with buffer supplemented with 1 mM DTT.

The Ibidi chip was then mounted to the stage of a Nikon TIRF microscope. Bilayers, Munc13 particles on bilayers, and vesicles attached to bilayers were respectively imaged at room temperature with the TIRF microscope using the corresponding laser.

### Counting Munc13 Copy Numbers.

To determine the number of copies of Munc13C in each DAG domain, we gradually bleached the image frames using suitable laser power at different positions. The bleaching profiles (particle fluorescence intensity versus time) were plotted and a variety of bleaching patterns were found. When the Munc13C copy number was small, the bleaching profile displayed apparent discrete steps, and the actual number of proteins can be determined from counting the number and intensity of steps. This method only works for relatively small copy numbers (generally, 5 to 6 or fewer) because the bleaching profile becomes smooth when the copy number is large. As an alternative for larger copy numbers, we fitted the intensity profile using:

[4] $$I\left(t\right)=I_{0}e^{-\frac{t}{\tau }}+B,$$

where $I\left(t\right)$ is the intensity at time t , $I_{0}$ is the initial intensity before bleaching, τ is the decay time constant, and B is the background. Hence, the copy number N can be obtained through:

[5] $$N=\frac{I_{0}}{i},$$

where i is the unit intensity of a single fluorophore, i.e., the average intensity of a single bleaching step determined using small clusters.

### Mass Photometry.

Mass photometry experiments were performed an OneMP instrument (Refeyn, Oxford, UK) at room temperature (43, 44). The microscope coverslips (24 × 50 mm, Fisher Scientific) and precut 2 × 2 silicon gasket wells (Sigma) were cleaned with MilliQ water, isopropanol, and dried with clean nitrogen flow and assembled with an adjustable cover-slip rack. The instrument was calibrated using β-Amylase and Thyroglobulin from Refeyn as protein standards: 18 μL of the buffer was added in an empty well on the coverslip and the laser was focused; then 2 μL of β-Amylase or Thyroglobulin was added and mixed by pipetting; data acquisition was started immediately and the MP video was recorded using the AcquireMP software, and then the software DiscoverMP was used to analyze the data and generate the calibration function. To measure the mass of the protein of interest, 10 μL buffer was added to an empty well on the coverslip, and then 10 μL of Munc13-1 solution at various concentrations (from 8 nM to 170 nM) was added and well mixed. Data collection was then started and the MP video was recorded. DiscoverMP was used to process the data (45). The distribution of molecular mass was plotted as histograms and fit with Gaussian peaks to obtain the average mass of different species and determine the molecular weight of Munc13-1.

### Lipid Membrane Preparation for CryoET.

Vesicles (DOPC/DOPS/PI(4,5)P_2_ in a molar ratio of 14/80/6) were prepared as described previously (24). Briefly, the lipid stocks were mixed in a chloroform with addition of 20 μL methanol to dissolve PI(4,5)P_2_, and the solvent was evaporated under N_2_ gas followed by vacuum drying for 1 h. The dried lipid film was rehydrated for 1 h at room temperature with constant vortexing in buffer containing 20 mM MOPS pH 7.4, 150 mM KCl, 1 mM EDTA, and 0.5 mM TCEP at a final lipid concentration of 1 mM. Next, the mixture was sonicated for 5 min using a bath sonicator (Branson Ultrasonics). Vesicles were used for crystallization next day after storage at 4 °C to sediment large lipid aggregates.

### Protein Crystallization for CryoET.

Vesicles were diluted down to lipid concentration of 100 µM and mixed with 1 μM Munc13C protein in a 1:1 (vol/vol) ratio. Once mixed, the samples were incubated at room temperature for 5 min and then kept on ice until freezing.

### Electron Microscopy Sample Preparation and Data Acquisition.

Samples (2.5 μL) were vitrified using a Vitrobot Mark IV (Thermo Fisher Scientific) held at 8 °C with 100% humidity. They were applied to freshly glow-discharged 200 mesh Lacey Formvar/carbon grids, and grids were blotted for 5 s with blot force −1 and then plunged frozen in liquid ethane cooled by liquid nitrogen. Samples with wild-type protein were imaged using Glacios Cryo TEM 200 kV (Thermo Fisher Scientific) equipped with a K2 Summit direct electron detector (Gatan). The K2 camera was used in counting mode, and detector dark and gain references were collected prior to each data acquisition session. The tilt-series movies were acquired using SerialEM with a bidirectional scheme starting from +20°; −43 to +44° tilt range with 3° increment, 80 e/A2 total dose. The nominal magnification was 13,500× resulting in a pixel size of 3.02 Å. Nominal defocus was set to −4.5 μm. Quadruple mutant tilt-series were collected using a 300 kV Titan Krios G2 transmission electron microscope (Thermo Fisher Scientific) equipped with a GIF Quantum LS energy filter and K3 Summit direct electron detector (Gatan). The slit width of the filter was set to 20 eV. Filter tuning was done using Digital Micrograph software (Gatan). The tilt-series movies were acquired using SerialEM at a nominal magnification of 42,000× (corresponding to a calibrated physical pixel size of 2.1 Å) using a dose symmetric scheme (46) with tilt range ± 51° and 3° increment. The tilt images were acquired as 11,520 × 8,184 super-resolution movies of 10 frames per each tilt corresponding to a tilt series total dose ∼120 e/Å2, respectively. Nominal defocus was set to −5 μm. Movies were aligned and saved as mrc stacks using alignframes from IMOD package (47). The resulting tilt series were aligned and reconstructed with 4x binning for and 8x binning by AreTomo (48), so the final pixel size of reconstructed tomograms was 12.08 Å and 16.8 Å. Topaz denoising was used to denoise selected tomograms using a unet-3d-20a pretrained model (34).

## Supplementary Material

Appendix 01 (PDF)

## Data Availability

All study data are included in the article and/or *SI Appendix*.
