# Supplementary material for: Diacylglycerol-dependent hexamers of the SNARE-assembling chaperone Munc13-1 cooperatively bind vesicles
Source: Proc Natl Acad Sci U S A. 2023 Oct 26;120(44):e2306086120. doi: 10.1073/pnas.2306086120 (PMC10623011; doi:10.1073/pnas.2306086120)
Supplement: Supplementary file 1 — Appendix 01 (PDF) [file pnas.2306086120.sapp.pdf]

PNAS

Supporting Information for

Diacylglycerol-dependent hexamers of the SNARE-assembling chaperone Munc13-1 cooperatively bind vesicles

Feng Li, Kirill Grushin, Jeff Coleman, Frederic Pincet, and James E. Rothman
Correspondence to James E. Rothman or Frederic Pincet
Email: james.rothman@yale.edu, frederic.pincet@ens.fr

This PDF file includes:

Supporting text
Figures S1 to S9
Tables S1
SI References

Supporting Information Text

Models of Munc13-1 binding to DAG microdomains

In the following models we will assume that the DAG microdomains are of uniform area which is reasonable considering the measured area distribution (see main text).

1. Isolated Munc13-1 that bind DAG microdomains independently

In this first model, we will assume that the clusters are just an optical bias due to the colocalization of isolated monomeric Munc13-1 molecules in the same DAG microdomain. According to the non-cooperativity of the Munc13-1 binding, the distribution of the number of Munc13-1 monomers, *i.e.*, the apparent cluster copy number in this model, should follow a Poisson probability:

$$P_M(m) = \frac{\langle m\rangle^m}{m!} e^{-\langle m\rangle} \quad \text{(S1)}$$

where m is the number of monomers and $\langle m\rangle$ the mean apparent cluster copy number. $\langle m\rangle$ cannot be directly measured experimentally because the domains without any Munc13-1 are not visible, *i.e.*, $P_M(0)$ is unknown. Hence, the observed copy number probability is:

$$P_{M_{obs}}(m) = \frac{P_M(m)}{1-P_M(0)} = \frac{\langle m\rangle^m}{m!}\frac{e^{-\langle m\rangle}}{1-e^{-\langle m\rangle}} \quad \text{(S2)}$$

Eq. 3 is valid only for $m \geq 1$. The mean of the observed cluster copy number distribution is therefore:

$$\langle m\rangle_{obs} = \frac{\langle m\rangle}{1-e^{-\langle m\rangle}} \quad \text{(S3)}$$

As expected, when $\langle m\rangle$ is large, it can very well be approximated by $\langle m\rangle_{obs}$ because there is hardly any DAG microdomain without Munc13-1 cluster.

Experimentally, we found $\langle m\rangle \approx \langle m\rangle_{obs}$ = 4.8 Munc13-1 per cluster. The predicted and observed cluster copy numbers are displayed in Fig. 2 and S2. They clearly do not match indicating that this model is not appropriate here.

2. Mixture of isolated Munc13-1 and *K*-mers

In this second model, we assume that Munc13-1 is present in two forms on the bilayer: monomers and *K*-mers. These two populations behave independently of each other and are randomly distributed among the cluster. Hence, each would present a copy number distribution following the Poisson probability, exactly as in the first model:

$$P_M(m) = \frac{\langle m\rangle^m}{m!} e^{-\langle m\rangle} \quad \text{(S4a)}$$

$$P_K(k) = \frac{\langle k\rangle^k}{k!} e^{-\langle k\rangle} \quad \text{(S4b)}$$

where m (resp. k) is the number of monomers (resp. *K*-mers) and $\langle m\rangle$ (resp. $\langle k\rangle$) the mean number of monomers (resp. *K*-mers) per cluster.

In principle, each cluster is a mix of monomers and pre-determined oligomers. In case of hexamers, the distribution of monomers and hexamers is exemplified in Supporting Table

S1. Hence the probability distribution $P_{Total}(N)$ of the cluster at size *N*, i.e., with *N* Munc13-1 molecules in the cluster, is:

$$P_{Total}(N) = \sum_{i=0}^{[N/K]} P_K(i)P_M(N - iK) = \sum_{i=0}^{[N/K]} \frac{\langle k\rangle^i}{i!} e^{-\langle k\rangle} \frac{\langle m\rangle^{N-iK}}{(N-iK)!} e^{-\langle m\rangle} \quad \text{(S5)}$$

$\langle m\rangle$ can be estimated exactly as in the first model by from the observed mean copy number obtained by only considering clusters with 1 to $(K - 1)$ copies of Munc13-1, $\langle m\rangle_{obs1-(K-1)}$, by rewriting Eq. (3):

$$\langle m\rangle_{obs1-(K-1)} = \frac{\sum_1^{(K-1)} mP_M(m)}{1-P_M(0)-\sum_K^{\infty} P_M(m)} \quad \text{(S6)}$$

$\langle m\rangle$ is then obtained numerically from Eq. S6.
Finally, $\langle k\rangle$ is well approximated by:

$$\langle k\rangle = \frac{\langle m\rangle_{obs}-\langle m\rangle}{K}$$

where <*k*> is the mean number of *K*-mers per cluster, and $\langle m\rangle_{obs}$ is obtained exactly as in the first model.
Once $\langle m\rangle$ and $\langle k\rangle$ are established the probability distribution described by Eq. S5 is completely defined.
When the *K*-mers are hexamers, the probability distribution obtained from Eq. S5 with these values for $\langle m\rangle$ and $\langle h\rangle$ is displayed in Fig. 2 and matches very well the experiment cluster copy number distribution.
Another prediction from this model is that the fraction of DAG microdomains without any Munc13-1 is:

$$P_{Total}(0) = e^{-\langle k\rangle}e^{-\langle m\rangle} = 0.06 = 6\% \quad \text{(S7)}$$

Experimentally we did not find any Munc13-1 molecule in 9% of DAG microdomains which is reasonably close to the predicted value considering the simplicity of the model.

3. Statistical comparison of the predicted and observed distribution of oligomers

To determine what oligomer seems to describe best the observed cluster size distribution, we use a parameter to test the oligomerization degree, *O,* that quantitatively compares the predicted and observed histograms for each K-mer

$$O = \sum_{m=1}^{m_{max}} \frac{P_{obs}(m)}{\left(P_{obs}(m)-P_{Total}(m)\right)^2} \quad \text{(S8)}$$

m_{max} was chosen as the largest cluster observed experimentally, 18. $P_{obs}(m)$ in the numerator is used to reduce the impact of the error on the less frequent cluster sizes and $\left(P_{obs}(m) - P_{Total}(m)\right)^2$ in the denominator directly represents the difference between the observation and the prediction. A larger O indicates a better approximation of the experimental distribution. For instance, in Fig. 2E (wild-type) and 3G (mutant), the best approximations are respectively obtained for hexamers and tetramers.

Fig. S1.

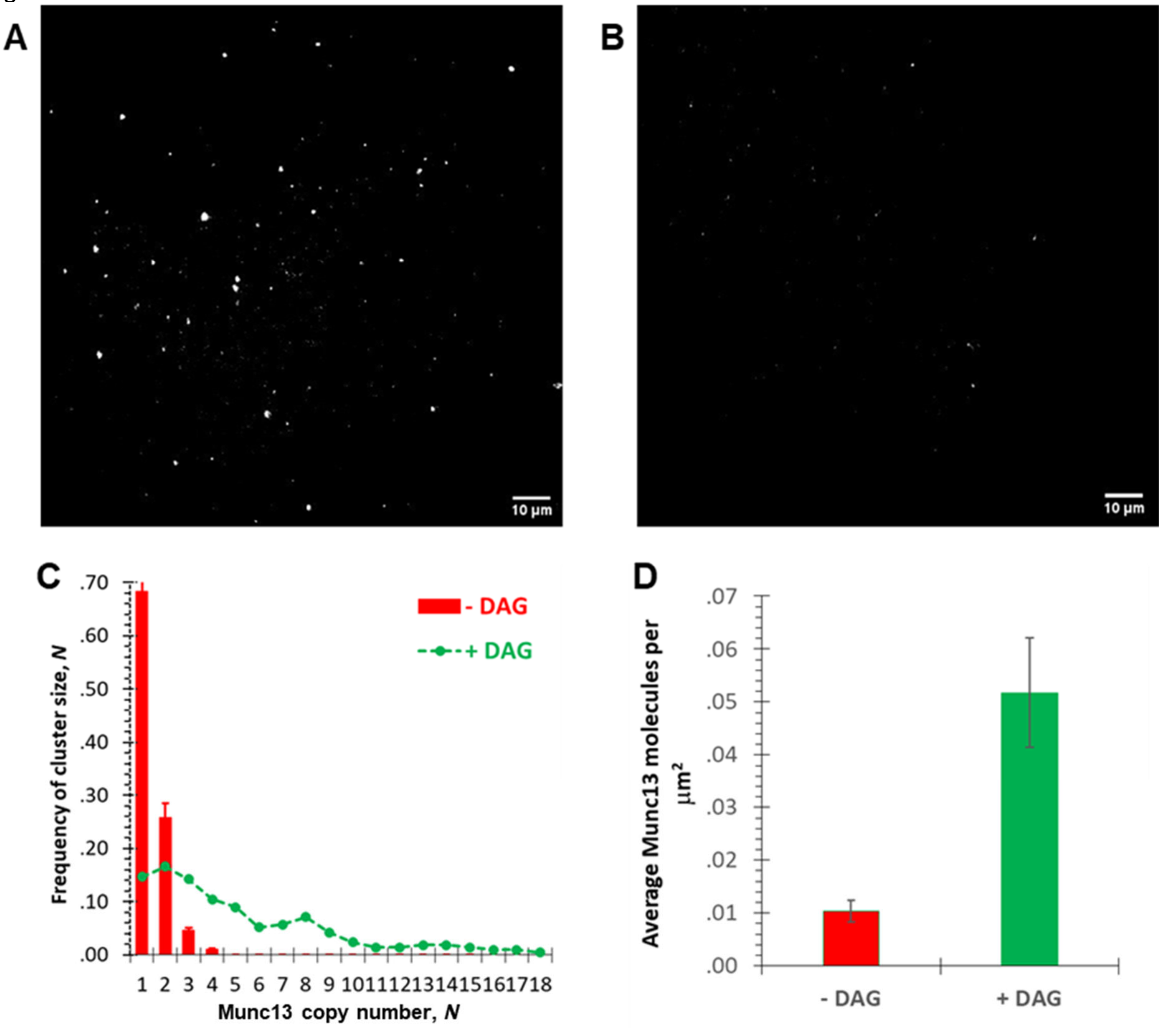

Supporting Figure S1. DAG plays a critical role in the formation of wildtype Munc13-1 clusters. (**A**) & (**B**) Representative TIRF image of wildtype Munc13-1 labeled with Alexa 488 on lipid bilayer membrane containing PC, PS and PIP2 in the presence (A) or absence (**B**) of DAG. (**C**) Distribution of the copy number of wildtype Munc13-1 molecules in the clusters on lipid bilayer in the absence of DAG (red columns). The green dashed line is the size distribution of clusters in the presence of DAG, which serves as a reference. (**D**) Comparison of the surface densities of wildtype Munc13-1 molecules on a bilayer in the presence or absence of DAG.

Fig. S2.

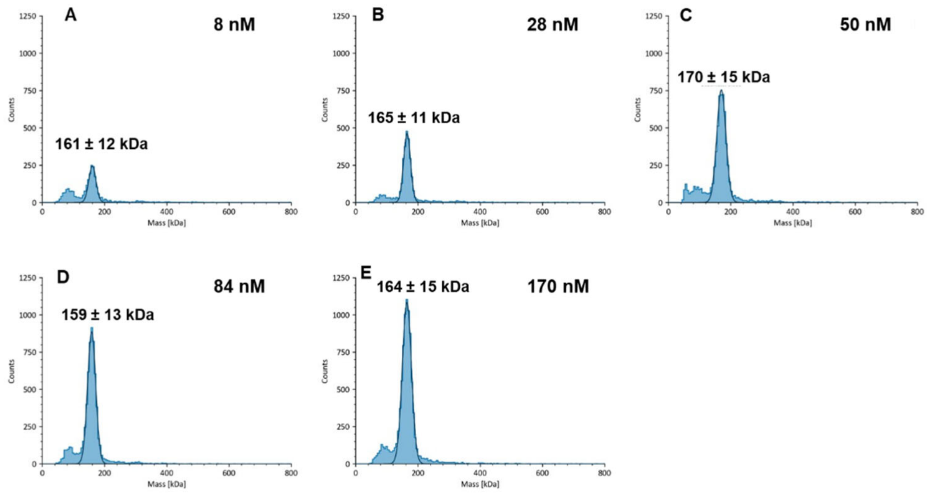

Supporting Figure S2. The molecular weights of Munc13C-Halo-Alexa 660 in solution at various concentrations were measured with Mass Photometer. The theoretical molecular weight of Munc13C-Halo-Alexa 660 is 167 kDa. Mass Photometry measurements were performed with various solution concentrations of Munc13C-Halo-Alexa 660: (**A**) 8 nM; (**B**) 28 nM; (**C**) 50 nM; (**D**) 84 nM; and (**E**) 170 nM. In each figure, the blue shaded area is the histogram that show the distribution of the molecular mass, and the solid line is a Gaussian fit of such distribution. No oligomer of Munc13C-Halo-Alexa 600 was found in solution for all the concentrations.

Fig. S3.

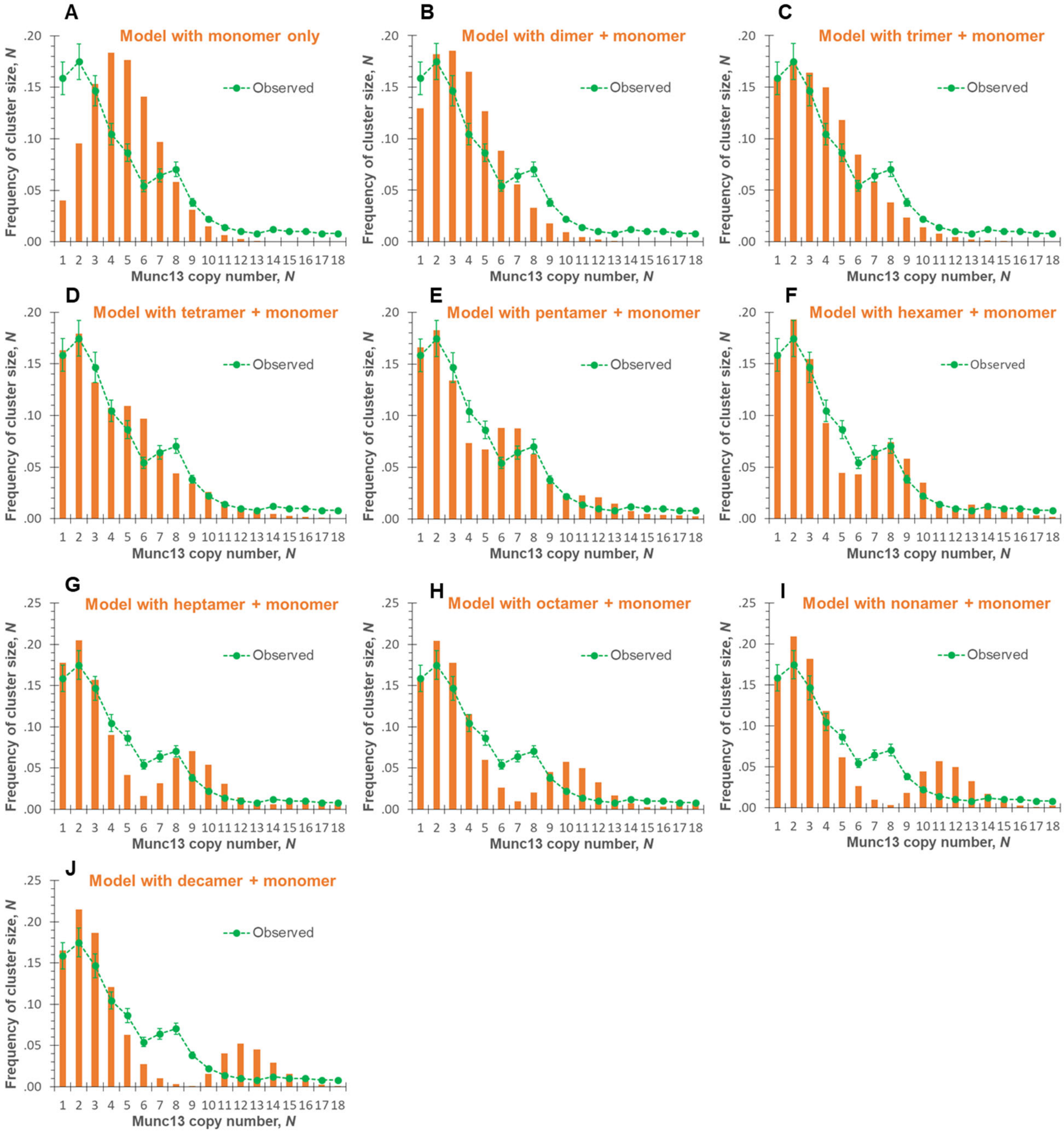

Supporting Figure S3. Poisson distribution modeling of oligomers in wildtype Munc13-1 clusters. (**A**) Single Poisson distribution, assuming all wildtype Munc13-1 in monomer state; (**B**) through (**J**) Dual Poisson distribution (monomer + uniform size oligomer), 2-step process: monomer with dimer (**B**), monomer with trimer (**C**), monomer with tetramer (**D**), monomer with pentamer (**E**), monomer with hexamer (**F**), monomer with heptamer (**G**), monomer with octamer (**H**), monomer with nonamer (**I**), and monomer with decamer (**J**). The orange bars represent predicted distribution of the copy number of Munc13-1 molecules in the clusters, and the green dashed lines are experimental data.

Fig. S4.

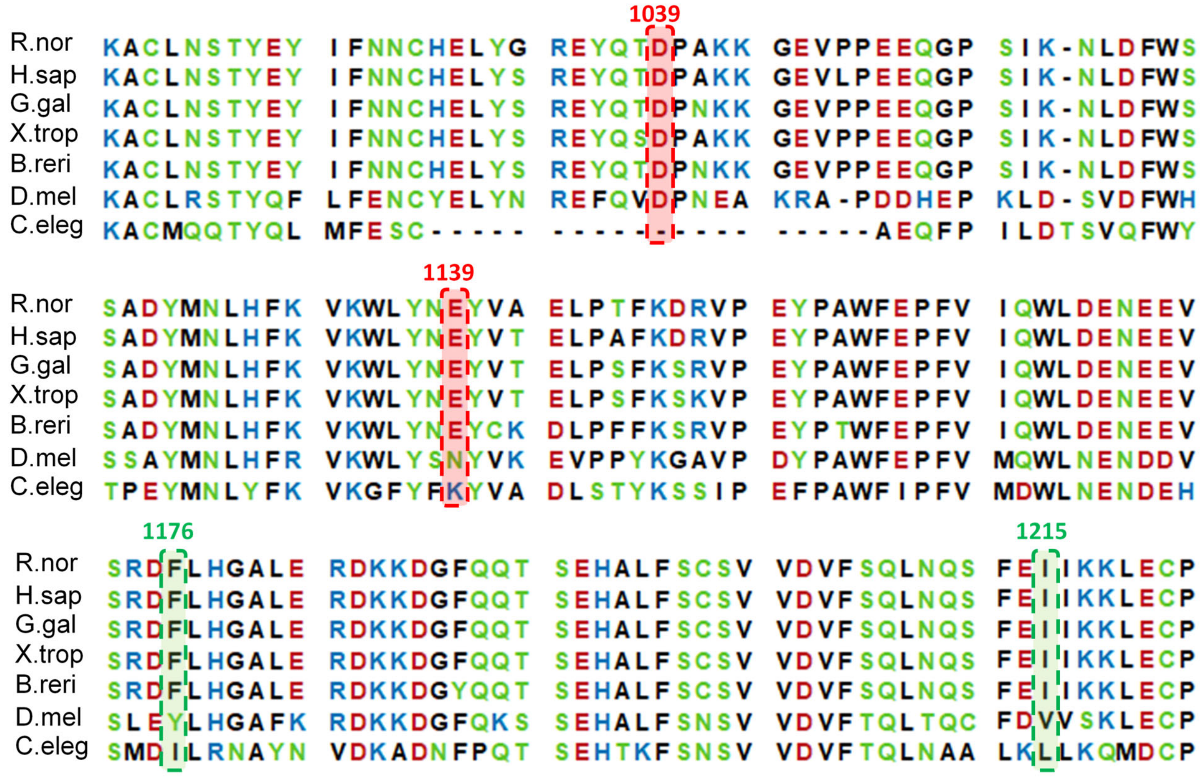

Supporting Figure S4. Sequence alignment of amino acid residues at the lateral interaction interface. Multi-sequence alignment of UNC13 homologs across species: five representatives of vertebrates (R.nor = Rattus norvegicus, H.sap = Homo sapiens, G.gal = Gallus gallus, X.trop = Xenopus tropicalis, B.reri = Brachydanio rerio) and two representatives of invertebrates (D.mel = Drosophila melanogaster, C.eleg = Caenorhabditis elegans). The numbers on the top are indicating residues positions in rat's Munc13-1 that were mutated and the corresponding aligned amino acid residues of other organisms are highlighted.

Fig. S5.

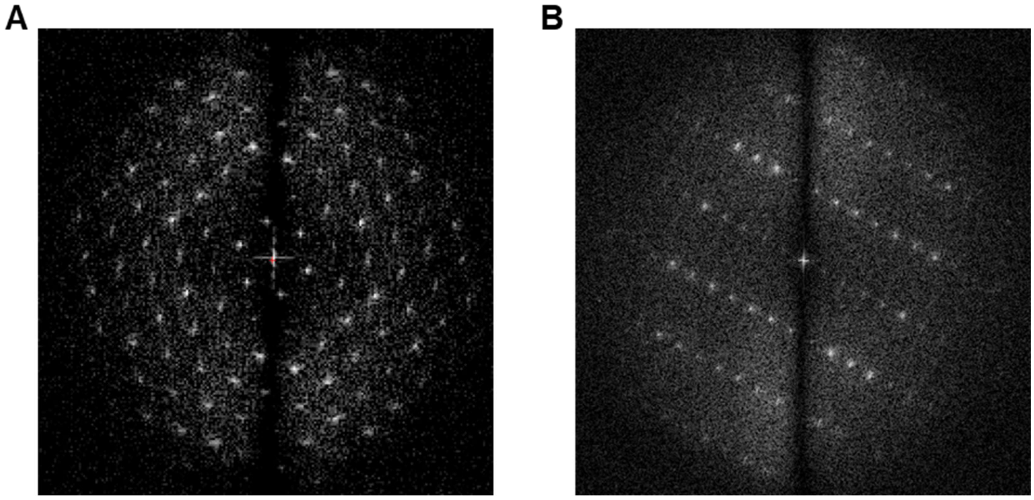

Supporting Figure S5. Diffraction pattern. Representative images of diffraction pattern (Fast Fourier Transform (FFT) pattern) of a 2D lattice between membrane bilayers (**A**) Wild-type Munc13-1, (**B**) F1176N/I1215N/E1139R/ D1039R Munc13-1 mutant. FFTs of the 2D crystal areas in reconstructed tomograms were obtained using 3dmod Slicer function (*1*).

Fig. S6.

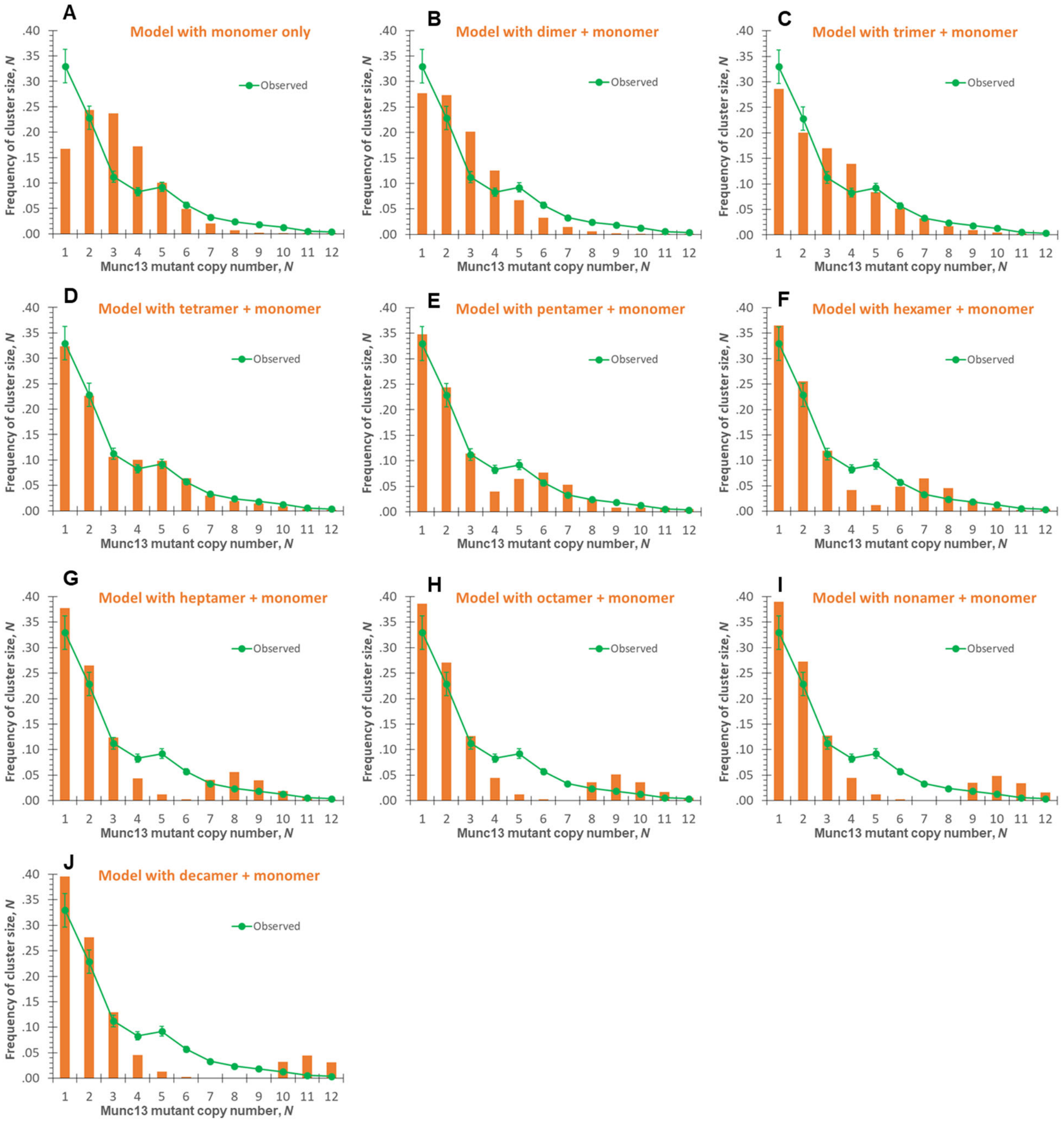

Supporting Figure S6. Poisson distribution modeling of oligomers in the clusters formed by Munc13-1 interface mutant, F1176N, I1215N, E1139R, and D1039R. (**A**) Single Poisson distribution, assuming all Munc13-1 mutant in monomer state; (**B**) through (**J**) Dual Poisson distribution (monomer + uniform size oligomer), 2-step process: monomer with dimer (**B**), monomer with trimer (**C**), monomer with tetramer (**D**), monomer with pentamer (**E**), monomer with hexamer (**F**), monomer with heptamer (**G**), monomer with octamer (**H**), monomer with nonamer (**I**), and monomer with decamer (**J**). The orange bars represent predicted distribution of the copy number of mutant molecules in the clusters, and the green dashed lines are experimental data.

Fig. S7.

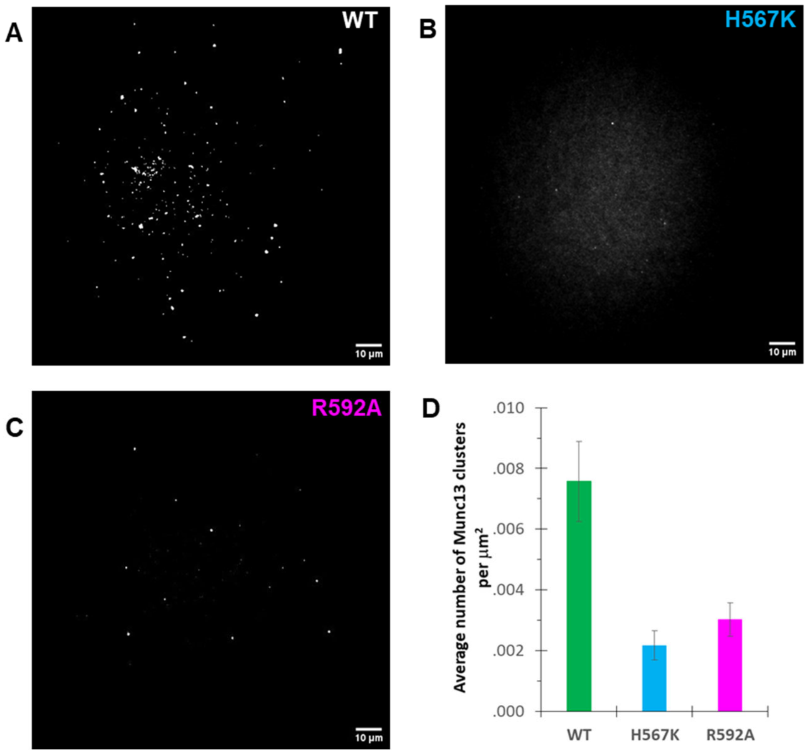

Supporting Figure S7. Mutations in DAG-binding motif within the C_1 domain reduce Munc13-1 recruitment to the lipid bilayer. (**A**) through (**C**) Representative TIRF images of: (**A**) wildtype Munc13-1; (**B**) Munc13-1 H567K mutant, which is known to prevent phorbol ester binding but also mimick the DAG-bound state (*2, 3*); and (**C**) Munc13-1 R592A mutant which was found to reduce the translocation of Munc13-1 to the membrane (*4*), on lipid bilayer membrane containing PC, PS, DAG, and PI(4,5)P_2. (**D**) Comparison of surface densities (number of particles per unit area) of wildtype Munc13-1, H567K mutant, and R592A mutant recruited to the lipid bilayers.

Fig. S8.

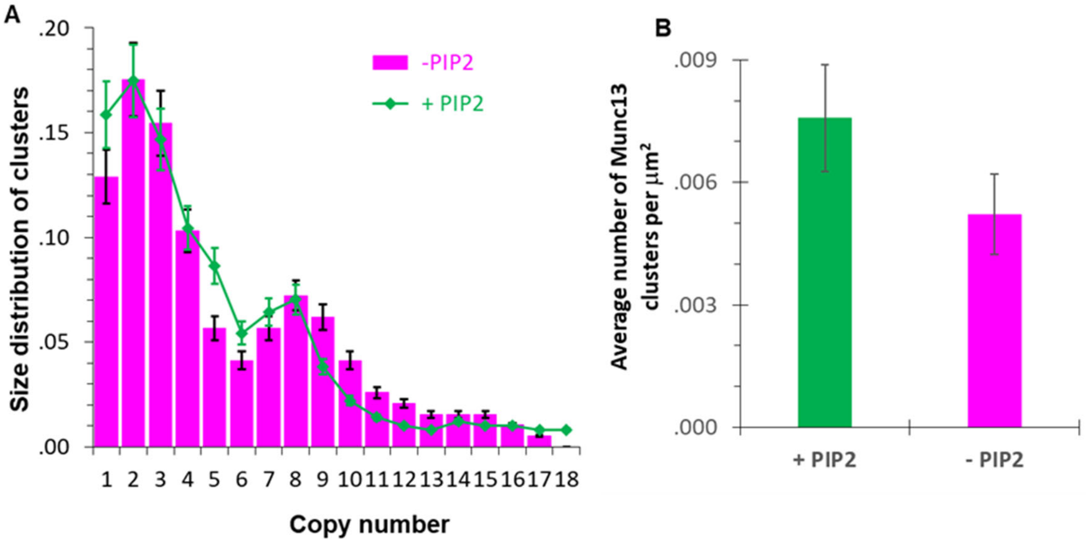

Supporting Figure S8. The omission of PI(4,5)P_2 from the lipid bilayer membrane slightly decreases Munc13-1 recruitment, but does not change copy number distribution of clusters. (A) Copy number distribution of clusters of wildtype Munc13-1 in the absence of PI(4,5)P_2 on lipid bilayer membrane containing PC, PS, and DAG (pink columns). The green dots represent the copy number distribution of wildtype Munc13-1 clusters in the presence of PI(4,5)P_2 (lipid bilayer containing PC, PS, DAG, and PI(4,5)P_2), which serves as a reference. Similar copy number distributions were observed for both cases. (B) Comparison of surface densities (number of particles per unit area) of wildtype Munc13-1 recruited to the lipid bilayers with or without PI(4,5)P_2.

Fig. S9.

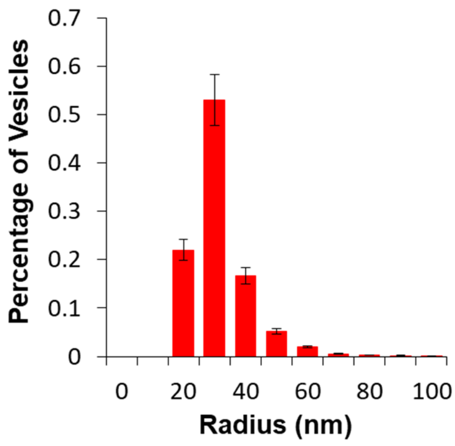

Supporting Figure S9. Size distribution of the vesicles used in the capture experiments. The hydrodynamic radii of the vesicles were measured by dynamic light scattering at room temperature. The size distribution was plotted as percentage of vesicles versus radius. The mean radius was 31 ± 9 nm.

Table S1.

**Munc13 cluster size**	**1**	**2**	**4**	**5**	**6**		**10**		**12**		
Monomer	1	2	4	5	6	0	10	4	12	6	0
hexamer	0	0	0	0	0	1	0	1	0	1	2

Table S1: the table is an example of the different possibilities to obtain clusters of various copy numbers by combining monomers and hexamers. For instance, a cluster with 10 Munc13-1 can be due to 10 monomers or 4 monomers and 1 hexamer.

SI References

1. J. R. Kremer, D. N. Mastronarde, J. R. McIntosh, Computer visualization of three-dimensional image data using IMOD. *J Struct Biol* **116**, 71-76 (1996).
2. F. Michelassi, H. Liu, Z. Hu, J. S. Dittman, A C1-C2 Module in Munc13 Inhibits Calcium-Dependent Neurotransmitter Release. *Neuron* **95**, 577-590 e575 (2017).
3. J. Basu, A. Betz, N. Brose, C. Rosenmund, Munc13-1 C1 domain activation lowers the energy barrier for synaptic vesicle fusion. *The Journal of neuroscience : the official journal of the Society for Neuroscience* **27**, 1200-1210 (2007).
4. Y. You, S. Katti, B. Yu, T. I. Igumenova, J. Das, Probing the Diacylglycerol Binding Site of Presynaptic Munc13-1. *Biochemistry* **60**, 1286-1298 (2021).
